# P-221. Impact of Stimulant Use on HIV and OUD: Five-Year Review on UAB’s OBAT Clinic

**DOI:** 10.1093/ofid/ofaf695.443

**Published:** 2026-01-11

**Authors:** Ann Harshfield, Ellen Eaton, Emma Kay, Leah J Leisch, Nicholas C Borgogna

**Affiliations:** University of Alabama at Birmingham, Birmingham, AL; University of Alabama, Birmingham, Birmingham, Alabama; University of Alabama at Birmingham, Birmingham, AL; University of Alabama at Birmingham, Birmingham, AL; University of Alabama at Birmingham, Birmingham, AL

## Abstract

**Background:**

Persons with HIV (PWH) with opioid use disorder (OUD) often face health disparities across the HIV care continuum, particularly when co-occurring stimulant use is present. Stimulant use has been associated with HIV viremia, lower retention in medication for opioid use disorder (MOUD), and increased risk for comorbid infections. This study aims to assess PWH presenting to an office-based addiction treatment (OBAT) clinic for PWH in the Deep South, focusing on the impact of stimulant use on treatment retention, HIV care outcomes, and healthcare utilization.

**Methods:**

We evaluated a clinic-based cohort at the University of Alabama at Birmingham HIV clinic from November 2019 to December 2024. We assessed baseline variables such as Hepatitis C status and diagnosis of co-occurring stimulant use disorder. Additionally, we compared health outcomes, comorbid disease, and retention in addiction care for persons with OUD with and without stimulant use over the five-year study period.

**Results:**

Of 113 individuals with OUD attending their initial OBAT visit, 102 were deemed appropriate for the clinic’s care. 84 (82%) individuals reported stimulant use or had a diagnosis of co-occurring stimulant use disorder. At the initial appointment, active Hepatitis C was more prevalent among individuals who used stimulants (29/84; 35%) versus those who did not (0/18; 0%). Undetectable HIV viral load at baseline was similar between groups: 43/83 (52%) for those who used stimulants and 9/18 (50%) for those who did not. STI prevalence was higher among individuals who used stimulants (32/67; 48%) than those who did not (3/11; 27%). Buprenorphine retention at 6 months was 51/57 (89%) for those who used stimulants and 16/17 (94%) for those who did not; at 12 months, 43/51 (84%) versus 11/12 (92%); at 2 years, 31/37 (84%) versus 11/11 (100%). Emergency room visits were higher among individuals using stimulants (71%) than those who did not (61%), while hospitalization rates were similar (39% vs 33%).

**Conclusion:**

Among PWH with OUD, a higher proportion of individuals who use stimulants were found to have Hepatitis C, STIs, and emergency room visits compared to those who do not use stimulants. However, both groups demonstrate high retention in buprenorphine treatment, highlighting the importance of integrated care.

**Disclosures:**

Ellen Eaton, MD, MPH, Gilead: Honoraria

